# P-661. Treatment Patterns Observed Among Immunocompromised And Frail Hospitalized COVID-19 Patients In The US

**DOI:** 10.1093/ofid/ofaf695.874

**Published:** 2026-01-11

**Authors:** Harriet Dickinson, Mark Berry, Amanda Kong, Alice Bersani, Isabella Lelis, Anand Chokkalingam

**Affiliations:** Gilead Sciences, Uxbridge, England, United Kingdom; Gilead Sciences, Inc., Foster City, CA; Aetion, New York, New York; Aetion, New York, New York; Aetion, New York, New York; Gilead Sciences, Uxbridge, England, United Kingdom

## Abstract

**Background:**

The treatment landscape for COVID-19 has evolved rapidly since the virus emerged in 2019. Immunocompromised, older and/or frail patients with COVID-19 are at higher risk of severe disease or death than the general population. This retrospective study examined treatment patterns in adults hospitalized with COVID-19 in the US from 12/1/2021 to 06/30/2024.

**Figure 1:**
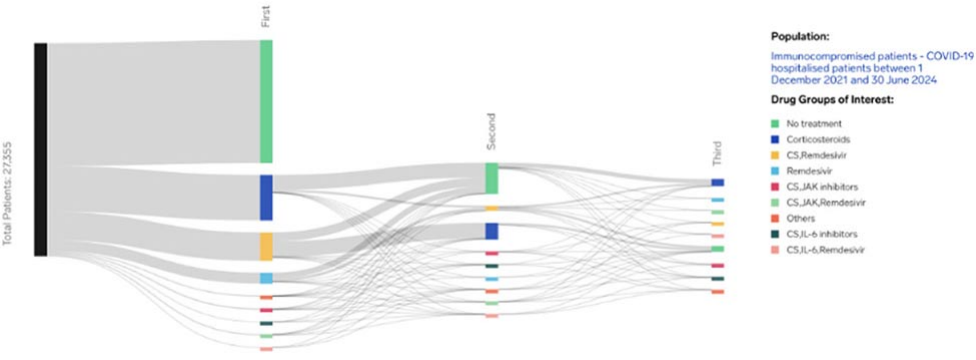
A Sankey Plot illustrating the treatment pathways experienced by immunocompromised hospitalised patients with COVID-19

**Methods:**

Using linked claims and chargemaster data, adults hospitalized with COVID-19 and >6 months of baseline healthcare insurance enrolment were selected. Corticosteroids (CS), remdesivir (RDV), Janus kinase inhibitor (JAKi), interleukin-6 inhibitors (IL-6i), nirmatrelvir plus ritonavir, and molnupiravir use were assessed from admission until death, discharge/transfer, 28 days after admission, or end of data. Therapies initiated prior to admission were not counted. Key subgroups were defined by age, diagnosis codes, and a published frailty algorithm.

**Results:**

The main cohort included 73,555 patients (mean age 59 years; 44% male). Most (59%) patients did not receive any COVID-19 treatments. The most common first-regimen treatments were CS alone (23%), CS/RDV combination (12%), and RDV alone (4%). Second regimen and third regimen treatments were identified in 13% (9,483) and 5% (3,984) of patients, respectively.

Among the 27,355 immunocompromised patients included (Figure 1), 58% had no treatments of interest. The most common initial treatment regimens were CS alone (21%), CS/RDV combination (13%), and RDV alone (5%). Second regimens were observed in 12% of immunocompromised patients; 5% had a third regimen treatment.

In patients without RDV-contraindicated conditions, RDV was used by 22% (3,765/16,775) of immunocompromised patients and 27% (4,928/18,316) of patients aged >65 during the assessment period. In patients aged >65, RDV was more commonly used in robust patients (28%), compared to mildly frail (24%) and moderate/severely frail (23%) patients.

**Conclusion:**

Many patients hospitalised with COVID-19 did not receive any COVID-19 indicated treatments. Less than 1 in 5 immunocompromised patients received RDV, indicating a potential treatment unmet need. Frail older patients were less likely to receive RDV than robust patients; further research is needed in this area.

**Disclosures:**

Harriet Dickinson, PhD, Gilead Sciences: Employment|Gilead Sciences: Stocks/Bonds (Public Company) Mark Berry, PhD, Gilead Sciences, Inc.: Employee|Gilead Sciences, Inc.: Stocks/Bonds (Public Company) Amanda Kong, DrPH, Aetion: Employee|Aetion: Stocks/Bonds (Private Company) Alice Bersani, Masters, Aetion: Employment|Aetion: Stocks/Bonds (Private Company)|Boehringer ingelheim: Employment Isabella Lelis, BA, Aetion: Employment Anand Chokkalingam, PhD, Gilead Sciences: Employment|Gilead Sciences: Stocks/Bonds (Public Company)

